# Supplementary material for: Phenotypic Detection of Hemin-Inducible Trimethoprim-Sulfamethoxazole Heteroresistance in Staphylococcus aureus
Source: Microbiol Spectr. 2021 Oct 27;9(2):e01510-21. doi: 10.1128/Spectrum.01510-21 (PMC8549745; doi:10.1128/Spectrum.01510-21)

Running title: monoclonal SXT heteroresistance *S. aureus*

Supplementary Appendix

Phenotypic detection of hemin-inducible trimethoprim-sulfamethoxazole hetero-resistance in *Staphylococcus aureus*

Dennis Nurjadi[1#], Quan Chanthalangsy[1], Elfi Zizmann[1], Vanessa Stuermer, Maximilian Moll[1], Sabrina Klein[1], Sébastien Boutin[1], Klaus Heeg[1], Philipp Zanger[1,2]

[1]Department of Infectious Diseases, Medical Microbiology and Hygiene, Heidelberg University Hospital, Im Neuenheimer Feld 324, 69120 Heidelberg, Germany

[2]Heidelberg Institute of Global Health, Heidelberg University Hospital, Im Neuenheimer Feld 130, 69120 Heidelberg, Germany

Table of contents

Supplementary Figure S1. The different phenotypic resistance growth characteristics on Mueller-Hinton Agar supplemented with 5% horse blood.......... 2

Supplementary Figure S2. Stability of heteroresistance phenotype.......... 3

Supplementary Figure S3. Photometric population analysis profile.......... 4

Supplementary Figure S4. Association between MLST and heteroresistance phenotype in *Staphylococcus aureus*.......... 5

Supplementary Figure S5. Auxotrophy testing.......... 6

Supplementary Figure S1. The different phenotypic resistance growth characteristics on Mueller-Hinton Agar supplemented with 5% horse blood.

D2201=fully resistant phenotype;KE7160=low-density heteroresistance phenotype; D2198=high-density heteroresistance phenotype; ATCC25923=fully susceptible phenotype

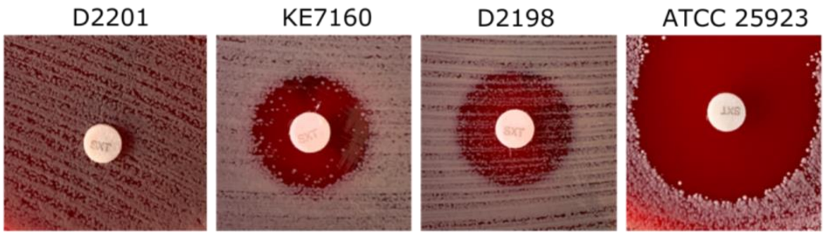

Supplementary Figure S2. Stability of heteroresistance phenotype.

Agar diffusion of a colony of trimethoprim-sulfamethoxazole resistant phenotype subcultured for 4 consecutive days exhibited similar heteroresistance phenotype as the original clone.

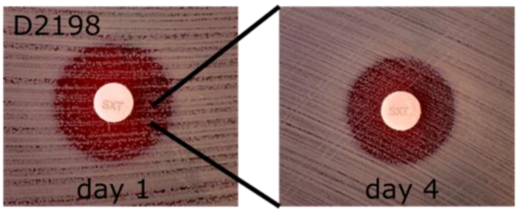

Supplementary Figure S3. Photometric population analysis profile.
Photometric growth analysis, measured at OD_{590nm} in various trimethoprim-sulfamethoxazole (1:19) concentrations in Mueller-Hinton broth without **(a)** and with 2.5 UI/ml thymidine phosphorylase **(b)**. SXT concentration is expressed as concentration of trimethoprim component. Abbreviations: TP=thymidine phosphorylase, SXT=trimethoprim-sulfamethoxazole

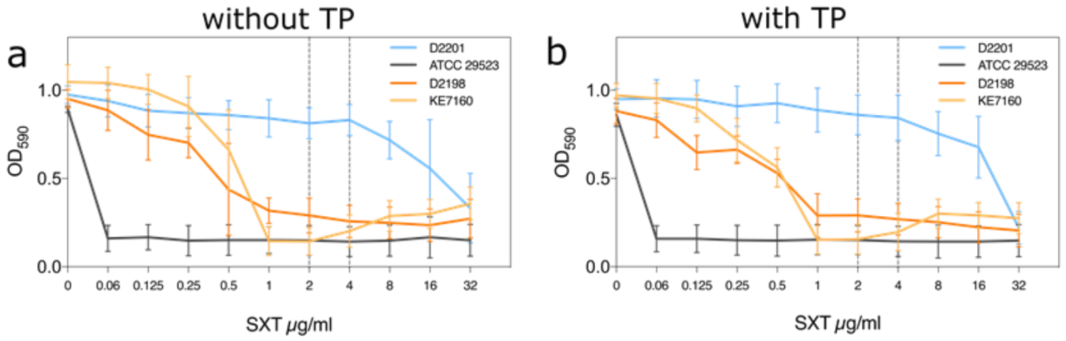

Supplementary Figure S4. Association between MLST and heteroresistance phenotype in *Staphylococcus aureus.*

The heteroresistant phenotype towards trimethoprim-sulfamethoxazole is not confined to a particular ST-group. Only MLST with ≥ 2 isolates (n=81/95) were included in this analysis. Two isolates with fully resistant phenotype were not included in the figure. Abbreviations: MLST=multi-locus sequence typing; ST=sequence type

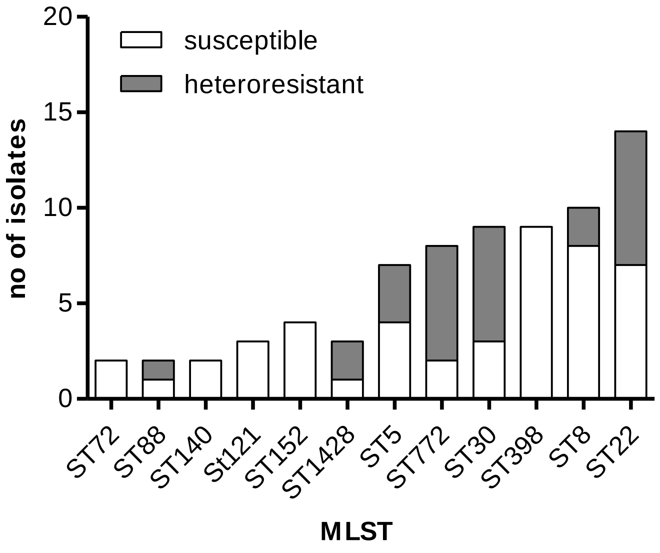

Supplementary Figure S5. Auxotrophy testing.

Staphylococcus aureus isolates D2198 and KE7160 did not exhibit thymidine, menadione or hemin auxotrophy. Both isolates exhibited normal growth on Mueller-Hinton agar and the presence of thymidine (T), menadione (M) or Hemin (X) did not change growth characteristics of the colony.

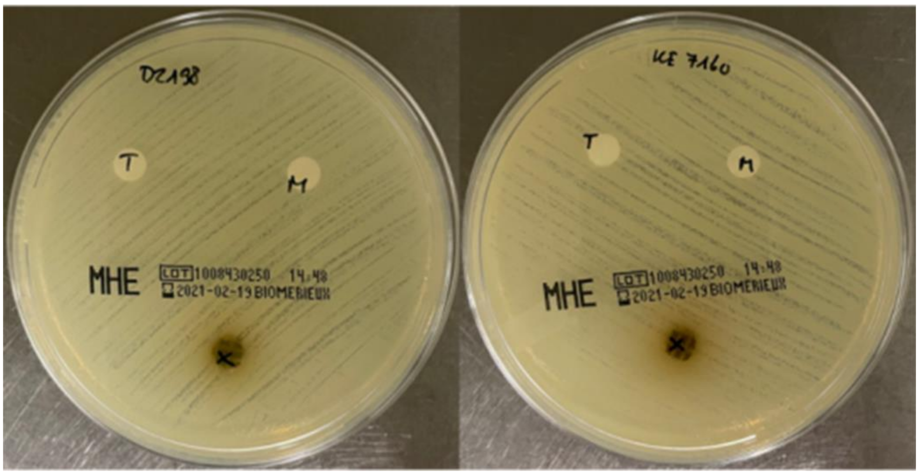

Supplement: SUPPLEMENTAL FILE 1 — Supplemental material. Download SPECTRUM01510-21_Supp_1_seq1.pdf, PDF file, 0.6 MB [file spectrum01510-21_supp_1_seq1.pdf]
